# Exploration factors on smoking among female adolescents based on the viewpoints of Iranian adolescent girls

**DOI:** 10.1186/s12905-022-01791-1

**Published:** 2022-06-01

**Authors:** Hadi Tehrani, Mehrsadat Mahdizadeh, Nooshin Peyman, Mahdi Gholian-Aval, Elham Charoghchian Khorasani, Alireza Jafari

**Affiliations:** 1 Social Determinants of Health Research Center, Mashhad University of Medical Sciences, Mashhad, Iran; 2 Department of Health Education and Health Promotion, School of Health, Mashhad University of Medical Sciences, Mashhad, Iran; 3 Department of Health Education and Health Promotion, School of Health, Social Development and Health Promotion Research Center, Gonabad University of Medical Sciences, Gonabad, Iran

**Keywords:** Tobacco, Cigarette, Female adolescents, Psychological factors, Qualitative study

## Abstract

**Background:**

In recent years, the prevalence of smoking in women has increased significantly. This study aimed to explain the effective factors in the tendency to smoke in female adolescents, employing a qualitative method of content analysis in Mashhad, Iran.

**Methods:**

The data for this conventional content analysis were obtained by conducting semi‑structured interviews with 20 female adolescent smokers. For open coding, integrating codes, creating main category and subcategories, and extracting quotation associated with codes and subcategories, MAXQDA software version 10 was used.

**Results:**

Based on the results of the study, one main category and six subcategories were identified to influence the decision to smoke. The main category was interconnection of psychological and gender experiences. The subcategories included self-management weakness, ignoring the side effects, gender orientation, negative psychological experiences, the attraction of cigarettes, and positive psychological experiences.

**Conclusion:**

Psychological and gender factors play an important role in initiating people's behavior and tendency to smoke and smoking prevention programs in educational settings need to focus efforts around what is known about young women smokers.

## Background

Tobacco use is a major public health issue around the world [1] and is a risk factor in a range of chronic and non-communicable disease (such as cardiovascular, respiratory, cancer, and stroke). Smoking is also one of the ten most significant causes of death in the world [2]. Seventy percent of the total tobacco consumption in the world is accounted for by developing countries [2]. In recent years, health plans in some developed countries have succeeded in reducing smoking rates, but in developing or low-income countries, smoking rates are on the rise at a young age [3–5]. The results of a global systematic review and Meta-analysis study showed that the current smoking rates in adult women and adolescent girls were 13% and 15%, respectively. The prevalence of current cigarette smoking is 21% in Oceania, 22% in America, 18% in Europe, 12% in Africa, and. 0.8% in Asia; an upward trend across all continents [6].

The causes of death associated with smoking include lung cancer (38%), ischemic heart disease (19%) and chronic obstructive pulmonary disease (12%). Smoking may shorten life expectancy by approximately two or three years for men and one year for women [1]. The results of a meta-analysis study of Iranian women showed that the prevalence of smoking was 0.7% to 25% [7]. Smoking among women is increasing and the rate of smoking among women is increasing faster than smoking among men [8]. Also, the use of hookah (pipe) has become fashionable among women in the Middle East, and this has also led to an increase in tobacco use [9, 10].

There are many reasons why teenagers smoke. The results of a recent study in Iran showed that from the perspective of teachers, parents, and students, the significant factors in smoking in adolescents were a feeling of importance in the adolescents who smoked and attracting the attention of others [11]. The results of a qualitative study on Iranian adolescents showed that the availability and cheap price of cigarettes was one of the most important factors in adolescents' tendency to smoke [12]. The results of a study on Korean adolescents showed that adolescents' tendency to take risks is one of the reasons for their tendency to smoke [13].

Most studies on smoking are related to its prevalence and fewer studies have been conducted on smoking behavior in female adolescents in Iran [14–16]. Lack knowledge in this field makes it difficult to design and implement effective prevention programs [17]. In Iran, because of the traditional cultural style that has already existed, some behaviors by women are not acceptable and there is no good social view of it. But in recent years, due to the cultural changes that have occurred due to the increase in global communication, some behaviors that were unsuitable before have changed and doing these behaviors are becoming normal social behaviors.

Smoking among Iranian women has been a taboo but this has decreased in recent years and Iranian women have become more inclined to smoke[18]. Most research has been done quantitatively and the prevalence has been investigated and the reasons for the tendency of women and girls, especially adolescent girls, have been less studied. To discover these reasons, the best research method is to conduct a qualitative research. This study hopes to extract points about the determinants of the onset and continuation of smoking in girls so that the findings can be used to redesign smoking prevention strategies to reduce smoking, particularly in girls. This study was conducted to exploration factors on smoking among female adolescents based on a qualitative method of content analysis. It took place in Mashhad, Iran.

## Methods

This study was conducted using qualitative conventional content analysis method to explain the process of starting smoking in adolescent girls (the age group was 13 to 19) in Mashhad, Iran. To collect information, we referred to parks, tobacco consumption places, coffee shops. Also, some participants entered the study through the snowball method. Also, some participants entered the study through the snowball method. Purposeful sampling was carried out and continued until the data was saturated. In qualitative research, the estimated sample size depends on many factors. Based on the results of a study, it is important to evaluate the sample size to achieve data saturation. Researchers should consider a number of factors, such as the quality of the data received, the scope and nature of the study, and the useful information obtained from each respondent [19–22].

Data analysis of 17 interviews revealed that the data was saturated in that no new codes emerged and themes were repeated. Three more interviews were subsequently conducted to ensure data saturation had been achieved. Twenty interviews were conducted in total. The text of the interviews were listened to several times to achieve a comprehensive understanding of participants' experiences. Inclusion criteria included at least one year of smoking, participant age of less than 20 years, informed consent by each participant (though a written consent form), Iranian citizenship, and being a resident of Mashhad. Adolescents who had difficulty sharing their experiences were excluded from the study.

In this study, data were collected through semi-structured interviews. Basic questions/guidance included “When did you start smoking?”, “Tell the story of the first time you smoked”, “What factors had the most impact on your tendency to smoke?”. At the end of each interview, the participants were thanked, and asked if they had any other comments to make. The contact information of the researcher was provided to the participants. The duration of each interview was between 30 and 70 min and place of the interviews were parks and coffee shops.

In this study, the data was analyzed by conventional content analysis based on the five steps of Graneheim and Lundman[23]. In the first step, the text of the recorded interview was transcribed and used as the main data for the research. In the second step, the audio tape was listened to several times, and the handwritten text was checked against this. At this stage, in order to understand the data, the researchers repeatedly familiarize themselves with the content. Then handwritten texts were divided into units of meaning. In the third step, the semantic units were coded. In other words, in this step, the participants' experiences were determined in the form of overt and covert concepts in the form of sentences or paragraphs and initial codes, then coding and abstraction were performed. In the fourth step, based on the continuous comparison of similarities, differences, and proportions, the codes that referred to a single topic were placed in a category. Then the sub-categories were categorized and the main categories determined. Ambiguities were addressed by the participants in subsequent interviews. In the last step, interpretive analysis, the central concept of each class was identified and the main abstract concepts extracted. The concepts were reviewed based on the explanation and interpretation of all the data. Once there was duplication of codes and a lack of new categories emerging, no further interviews were conducted. For open coding, integrating codes, creating subcategories, and extracting quotations associated with codes and subcategories, MAXQDA software version 10 was used.

Dependability, credibility, transferability, and confirmability used to ensure the trustworthiness of the study[24]. To ensure credibility, the researchers spent a lot of time conducting the interviews and analyzing the data. Also, the collected data were implemented and coded immediately after each interview. For transferability, the researchers provided a detailed description of the participants and the overall analysis. Confirmability and transferability were maintained by returning the codes extracted from the interviews to the participants and receiving their views[24]. In addition, the research team discussed on the findings at various stages of the analysis.

## Results

The results of this study were obtained through semi-structured interviews with twenty female adolescent smokers. In this study, the mean (standard deviation) of the participants was 16.30 (± 2.70). Based on the results of demographic characteristic, 10% (n = 2) had 2 brothers, 20% (n = 4) had one brother, 5% (n = 1) had two sisters, 10% (n = 10) had one sister, 5% (n = 1) had three sisters, and 35% (n = 7) were single children. Based on the results obtained in response to the overall research question of explaining the effective factors in the tendency of female adolescents to smoke, one category and six sub-categories were extracted. More complete results can be seen in Table 1.

**Table 1:** The view of adolescent female smokers about personality factors influencing their tendency to smoke

Maine category	Sub-category	Open codes
Interconnection of Psychological and Gender experiences	Self-management weakness	Lack of power to say no
		The most important of the present time
		Poor network of friendships
		Carrying a cigarette in bag
	Ignoring the side effects	Ignoring the negative impact of smoking on health
		Ignoring the harms due to pleasure
		Acceptance of smoking side effects
		Ignoring the consequences
	Gender orientation	The search for female identity
		Prove your sense of independence to others
		Feminine restrictions
		Belief in gender equality
		Repression of girls in society
		Not accepting your gender
		Tendency to the male gender
	Negative psychological experiences	Anxiety problem
		Feeling empty
		Dissatisfaction with life
		To be disappointed
		Stress
		Feeling depressed
		Feelings of loneliness
		Forced to marry at a young age
		Beating experience
		Sexual abuse
		Tobacco / hookah experience
		Cigarette dependence
		There was no daily calm
		Facing smoking in the family
		Emotional failure
	Positive psychological experiences	Feelings of pleasure
		Sense of curiosity
		Experience the search for peace
		The pleasure of experiencing different things
		Excitement
		Enjoy the experience of smoking other tobacco
		Distract
		Getting amused
		Psychological evacuation
	Attraction of cigarette	Easy cigarette smoking
		The taste of cigarettes
		The pleasure of smelling cigarettes
		Flavored cigarettes
		Colored cigarettes
		Loving the style of smoking

### Main category: interconnection of psychological and gender experiences

#### First sub-category: *Self-management weakness*

In this sub-category, participants stated that factors such as the importance of the present moment, weakness in foresight, having cigarettes with them, a lack of power to “Say No” to peers, and a particular network of friendships, appeared to increase the tendency to smoke.

… If I had a close friend who told me not to smoke anymore, I would not smoke or quit. But because all my friends smoke, I do too (Interview 12, age 19).

… I always carry a pack of cigarettes in my bag for consumption (Interview 9, age 18).

… one of my friends who we have been friends with for 5 or 6 years, I knew in my school that he smokes, he gave me a cigarette butt and I tried (Interview 10, age 17).

#### Second sub-category: *Ignoring the side effects*

In this sub-category, participants stated that ignoring the negative impact of smoking on their health and accepting the side effects of smoking, are effective factors in the tendency of female adolescents to smoke.

… I get together with my friends and smoke, I tell myself … a thousand people are smoking and my grandfather is smoking and is 80 years old and living a healthy life (Interview 4, age 16).

… So for me the impact that smoking has on my health, it does not matter (Interview 14, age 18).

… Everyone who smokes knows about its side effects. I know, and it is written on every pack of cigarettes that smoking causes lung cancer (Interview 7, age 16).

#### Third sub-category: gender orientation

In this sub-category, participants stated that factors such as discovering their feminine identity, restrictions on women/girls in society, challenging these and belief in gender equality are all factors in the tendency to smoke.

… Men smoke very easily in society. But if a woman wants to smoke, she should smoke somewhere so that others do not see her. If smoking is not good, there is no difference between men and women. So if I want to smoke somewhere, I go and stand next to a man who smokes and I smoke because I am no different from him (Interview 7, age 16).

… Sometimes I smoked because it didn't matter what other people thought of me, and I wanted to at least prove to myself that and others are not allowed to decide for me (Interview 12, age 19).

#### Fourth sub-category: negative psychological experiences

In this sub-category, participants stated that factors such as smoking dependence, lack of daily calm, anxiety, feeling empty, dissatisfaction with life, frustration, stress, feelings of depression, feelings of loneliness, unpleasant events, experience of punishment by parents at an early age, sexual abuse, previous experience of smoking and emotional failure are effective in their tendency to smoke.

… When I first started smoking, I was not in a good mood and I was depressed, and this encouraged me to smoke (Interview 17, age 18).

… When I'm nervous, I just think about smoking. I tried different ways to reduce my discomfort, but I did not succeed. That means I smoke a lot more than usual when I'm nervous (Interview 16, age 15). … When I started smoking a lot, my … relationship ended after 4 years and I was under a lot of stress, and at that time my smoking was very high (Interview 13, age 16).

… I had a friend commit suicide about a year ago and I was very upset, so I smoked a lot at that time (Interview 10, age 17).

#### Fifth sub-category: positive psychological experiences

In this sub-category, participants stated that factors such as feelings of pleasure, curiosity, searching for relaxation, the pleasure of experiencing different things, excitement and mental discharge, are effective in their tendency to smoke.

… Every time I smoke and ask smokers why they smoke, they say it is soothing. For example, half of my friends say the same thing and say that smoking helps to calm down (Interview 16, age 15).

… When I'm not feeling well and I'm nervous, I prefer to calm myself down with a cigarette (Interview 11, age 15).

… The first time I smoked was out of curiosity, and then I liked smoking and continued to use it (Interview 12, age 19).

…. I was thinking about how I could be happy and experience something new that feels good. I always thought I needed a lot more fun and relaxation in my life and the most accessible thing was this cigarette for me (Interview 14, age 18).

#### Sixth sub-category: attraction of cigarettes

The attractiveness of cigarettes is related to their appearance and intrinsic features. In this sub-category, participants stated that factors such as easy cigarette consumption, the good taste of cigarettes, the pleasure of the smell of cigarettes, flavored cigarettes, colored cigarettes, and the ‘style’ suggested by smoking are effective in the tendency of adolescent girls to smoke.

… Most women smoke these flavored cigarettes. Because other cigarettes are bitter, but these flavored cigarettes are not bitter and are in line with women's tastes (Interview 8, age 15).

… I saw in the society that smokers consume cigarettes in a beautiful style. Well, I found these to be very attractive and beautiful, and this made me gradually start smoking (Interview 9, age 18).

… I like that style of smoking, when I hold a cigarette in my hand I like its style (Interview 6, age 15).

## Discussion

This study aimed to explain the factors influencing the tendency to smoke in female adolescents based on a qualitative method of content analysis. Based on the results, six sub-categories of self-management weakness, inattention to complications, gender orientation, negative psychological experiences, the attraction of cigarettes, and positive psychological experiences were identified as the most important factors influencing adolescent girls' tendency to smoke.

### Self-management weakness

A common factor in adolescents' tendency to smoke was their self-management weakness. Most of the participants stated that they could not say no to the suggestion of friends to smoke. Some participants always carried a pack of cigarettes in their bags. Some people also stated that they had a weak network of friends and that loneliness was a factor in their tendency to smoke. Adolescent girls who feel self-managed and control their performance are reluctant to smoke because they are able to resist the temptation to smoke.

According to the results, most adolescents start smoking under the influence of their peers, and adolescents with self-management ability are more resistant to the temptations suggested by friends, and the likelihood of smoking is reduced [25]. Holding educational programs in schools can significantly increase adolescents’ skills in “Saying No” and protect them from the temptations of smoking [26, 27]. The results of other studies have shown that self-management and smoking are related, and self-management skills are effective in reducing smoking [28]. Self-management skills are one of the factors that lead a person to adopt a set of strategies to do or not to do behaviors. In addition, improving self-management skills is related to reducing tobacco use [29]. It seems that parents can play an important role in increasing adolescent self-management skills and in teaching their children to prepare to cope with peer pressure.

### Ignoring the side effects

In this study, most of the participants were aware of the side effects of smoking, but this knowledge did not prevent them from smoking. The results of a 2014 study showed that most smokers had little knowledge about most of the ingredients of tobacco [30]. Some people do not consider themselves to be smokers and this makes them think that smoking will not be dangerous for them [31]. Contrary to other study results, it seems that here, other factors, such as cigarette attractiveness, peer pressure, gaining pleasure, reducing stress and so on, play a more important role in smoking among adolescents who ignore the dangers of smoking.

Also, smokers may ignore the long-term potential consequences of smoking (such as addiction and lung cancer) in favor of the immediate pleasure they experience [32]. The results of a 2017 study showed that although smokers were aware of the side effects of smoking, they did not take these risks seriously and continued to smoke [33]. The negligence of the participants in this study may be due to two reasons. The first reason is that they may not have enough information about smoking and its side effects. The second reason for their inattention can be due to the immediate enjoyment of smoking, which has led them to deny the side effects and consequences of smoking.

### Gender orientation

Based on the results of this study, most participants believed in gender equality and believed that they, like men, should be able smoke freely. Some participants described smoking as a form of protest against gender inequality in society. Some people stated that the reasons for smoking were to prove their sense of independence and decision-making powers. The results of a 2016 qualitative study among Iranian women showed that social disobedience, self-expression, sense of independence, and symbols of gender equality were effective reasons for their tendency to smoke [34]. It seems that part of the tendency of Iranian girls to smoke was due to restrictions imposed on women in Iran. In Iranian culture, smoking is not regarded as a socially acceptable behavior, and there is a negative attitude and social stigma towards it. It was traditionally taboo in Iran and the number of female smokers was low. But now this culture has changed and female adolescents tend to smoke to demonstrate their freedom in decision-making and their belief in gender equality. They also believe that it is normal behavior for women to smoke like men.

### Negative psychological experiences

Most of the people in the study said that because of the conditions and problems in their lives, they use cigarettes as a painkiller for peace of mind and relaxation. Their problems included anger, stress, emotional problems, unpleasant experiences, feelings of emptiness, and so on.

The results of a similar study showed that adolescents often use smoking to relax, and relieve pain and discomfort [11]. The results of a study of Korean adolescents showed it was use to relieve stress [13]. The results of a study of adolescents in Greece showed that emotional and behavioral problems are a significant factor in adolescent smoking [35]. Adolescents seem to use smoking to solve their problems and discomforts and their other problem-solving skills appear to be impaired. Therefore, it is necessary to help young people develop personal skills to resist the acceptance of cigarettes.

### Attraction of cigarettes

One of the reasons for the propensity of female adolescents to smoke is the attractiveness of smoking. Study participants stated that smoking cigarettes is easier than using other tobacco products. They also said that smoking has a ‘beautiful’ style that makes them feel more attractive. In addition, the aesthetic aspects of cigarettes (such as their color and design) and the production of cigarettes according to people's different tastes (flavored cigarettes, cigarette smell, etc.) are factors in their smoking propensity.

The results of a qualitative study on Finnish adolescents' perceptions of cigarette packaging showed that simple cigarette packaging was less attractive to them. In addition, due to the simplicity of cigarette packs, health warnings have a more powerful effect in attracting the attention of young people. Simple cigarette packaging can be a powerful tool to reduce the number of youth smokers [36]. Attractive packaging with pleasing colors and designs increases people's tendency to smoke [11].

### Positive psychological experiences

In addition to the negative experiences that were effective factors in the tendency to smoke, in the present study, positive psychological experiences were also effective factors in the tendency to smoke. Most of the people in this study stated that they tended to smoke for pleasure, the pleasure of experiencing, having fun, curiosity, and reducing mental exhaustion.

A study by Chezhian et al. showed that environmental factors (62%), recreation (50%), and stress (48%) were among the main reasons reported by participants for starting to smoke [37]. A qualitative study on Iranian women showed that curiosity, personal interest and desire, and improved mood were effective in their tendency to smoke [34]. The pleasure of smoking is one of the reasons why smokers are reluctant to reduce or quit smoking and a factor in their failure to quit [38].

### Strengths and limitations

Certain limitations of this study may inhibit our ability to generalize from our findings. Our findings may not apply to all adolescent girls or girls outside of Iran. Another of the limitations of this study was that it did not assess respondents' prior knowledge of the risks of smoking. Finally, this study was based on a sample female adolescents so no comparison can be made with male adolescents. It is suggested that in future studies, the reasons for smoking in adolescent girls are also explained from the perspective of parents, teachers and policy makers. One of the strengths of this study was the discovery of the reasons for girls' tendency to smoke and pay attention to the cultural and social context for discovering these reasons. Another strength of this study was to investigate the reasons for the tendency to smoking in Iranian girls.

## Conclusion

Our findings show that there is a critical need for a deeper understanding of the experiences and needs of the girls. Based on the results, factors such as self-management weakness, ignoring the side effects, gender orientation, negative psychological experiences, the attraction of cigarettes, and positive psychological experiences were the most important reasons for the tendency of female adolescents to smoke. Due to the increasing prevalence of smoking among female adolescents, it is necessary to design and implement appropriate prevention programs for this age group. These programs should focus on exploring and addressing the social influences which encourage smoking. It is also necessary to pay attention to the role of personality factors in educational programs related to smoking prevention, which play an important role in the tendency of people to start to smoke.

## Data Availability

The data sets used and/or analyzed during the current study was available from the corresponding author on reasonable request.
